# Medical and Social Problems in the Scottish Highlands

**Published:** 1906-11-03

**Authors:** 


### Medical and Social Problems in the Scottish Highlands.

To the overburdened ratepayer of a town or city borough, oppressed with the anxiety and expense of his own municipal concerns, the idyllic peace and content of the country-side may appear as a delightful escape from the cries of the social reformer and the prickings of the civic conscience. Here, at all events, it may be judged, there are none of those acute questions which perplex and darken the public life and responsibilities of our great cities. Such a conclusion would probably be dispelled by experience, and those who doubt this statement must be readily convinced by a pamphlet which has recently been issued by Dr. Lachlan Grant, of Ballachulish, and entitled "Modern Highland Problems." Dr. Grant is an earnest advocate of many social changes the political aspects of which cannot engage us here. It may, however, be a not unprofitable method of spending a few moments to contemplate, amidst our own difficulties, the problems which present themselves to one of our *confrères* in the remote solitudes of the Scottish Highlands.

On the question of medical attendance Dr. Grant alleges that many of the Highland districts are most insufficiently supplied, and contends that this must necessarily be the case under the present system. His solution is the creation of a State medical service, and the appearance of a medical practitioner as a public servant in every district of the country. He would have, indeed, a public medical service exactly analogous to that of the Post Office, and just as the loss on the latter in some districts is compensated for by gain in others, so with the provision of medical assistance—the well-to-do sections of the country should pay for the poorer parts. In the same fashion Dr. Grant calls for the wider distribution of clinical laboratories and hospitals, which in his view are under existing arrangements far too much gathered into a few large centres. He would place all hospitals, infirmaries, and nursing homes under the rating authorities, and he condemns spasmodic charity as unworthy of a self-respecting and common-sense people.

Sanitation is another question which, even in the Highlands it seems, is in an unsatisfactory position. Houses exist, strange as it sounds to the crowded town-dweller, which are described as a disgrace to civilisation and as unfit for human habitation. Even when the law in its present form is sufficient to condemn this it is often not put into operation, as private and public influences oppose the recommendations of the local medical officers of health, and the necessary expense is sometimes sufficient to prevent improvement. All this Dr. Grant meets by a demand for further legislation, and by a claim that the difficulties of the Highlands shall be aided by contributions from the national exchequer. On somewhat similar principles Dr. Grant urges the provision of "suitable meals, conveyances, boots, shoes, and overcoats" for children who in order to attend school have to walk many miles daily, and for all school-children he would provide efficient medical examination and inspection. Thus the demands of many of our Socialistic friends in the cities find their endorsement north of the Grampians. It were idle to pretend that we are able to assent to all these propositions. But they are advanced by one who has made a careful study of the problems at issue, and they demand courteous consideration as the outcome of direct and first-hand knowledge.

A less contentious question discussed by Dr. Grant is that of afforestation. He holds that in many large areas in the Highlands timber could be grown not only with advantage to the natural aspect and climatic conditions of the country, but also as a profitable commercial undertaking. Quoting statistics from various authorities, he argues that not less than £18,000,000 of timber annually imported could be quite well grown in the Scottish Highlands. This, of course, would mean large opportunities for employment in country districts, a check on the rush into the large towns, and a diminution of the stream of emigration which takes regularly so much bone and sinew out of the country. Such are some of the social, economic, and sanitary problems of the Highlands of Scotland, and, however much we may differ from the solutions suggested, Dr. Grant's contribution is one of much interest and importance.

